# Relationship between growth and illness, enteropathogens and dietary intakes in the first 2 years of life: findings from the MAL-ED birth cohort study

**DOI:** 10.1136/bmjgh-2017-000370

**Published:** 2017-12-28

**Authors:** MAL-ED Network Investigators, Angel Mendez Acosta

**Keywords:** child health, nutritional and metabolic disorders, cohort study, environmental health, pneumonia

## Abstract

**Background:**

Dietary and illness factors affect risk of growth faltering; the role of enteropathogens is less clear. As part of the Etiology, Risk Factors and Interactions of Enteric Infections and Malnutrition and the Consequences for Child Health and Development (MAL-ED) study, we quantify the effects of enteropathogen infection, diarrhoea and diet on child growth.

**Methods:**

Newborns were enrolled and followed until 24 months. Length and weight were assessed monthly. Illnesses and breastfeeding practices were documented biweekly; from 9 to 24 months, non-breast milk intakes were quantified monthly. Routinely collected non-diarrhoeal stools were analysed for a broad array of enteropathogens. A linear piecewise spline model was used to quantify associations of each factor with growth velocity in seven of eight MAL-ED sites; cumulative effects on attained size at 24 months were estimated for mean, low (10th percentile) and high (90th percentile) exposure levels. Additionally, the six most prevalent enteropathogens were evaluated for their effects on growth.

**Results:**

Diarrhoea did not have a statistically significant effect on growth. Children with high enteropathogen exposure were estimated to be 1.21±0.33 cm (p<0.001; 0.39 length for age (LAZ)) shorter and 0.08±0.15 kg (p=0.60; 0.08 weight-for-age (WAZ)) lighter at 24 months, on average, than children with low exposure. *Campylobacter* and enteroaggregative*Escherichia coli* detections were associated with deficits of 0.83±0.33 and 0.85±0.31 cm in length (p=0.011 and 0.001) and 0.22±0.15 and 0.09±0.14 kg in weight (p=0.14 and 0.52), respectively. Children with low energy intakes and protein density were estimated to be 1.39±0.33 cm (p<0.001; 0.42 LAZ) shorter and 0.81±0.15 kg (p<0.001; 0.65 WAZ) lighter at 24 months than those with high intakes.

**Conclusions:**

Reducing enteropathogen burden and improving energy and protein density of complementary foods could reduce stunting.

**Key questions**

**What is already known about this topic?**

- Dietary inadequacy and high illness burden are well-established risk factors of poor childhood growth.
- More recent research has suggested that exposure to some enteropathogens in the absence of diarrhoea may additionally adversely impact growth.

**What are the new findings?**

- Higher enteropathogen burdens in non-diarrhoea stools, as well as lower energy and protein density of complementary foods, were found to be associated with poorer growth in Etiology, Risk Factors and Interactions of Enteric Infections and Malnutrition and the Consequences for Child Health and Development (MAL-ED) children with accumulated impacts on weight and length at 24 months.
- Whereas diarrhoea has previously been identified as a major driver of poor growth in developing countries, this analysis of MAL-ED children did not find diarrhoea to be a major risk factor for poor growth across the sites.

**Recommendations for policy**

- With continued efforts to prevent disease and improve infant and complementary feeding, specific interventions are needed to address cryptic enteropathogen burden.
- The work here suggests modifying the long-standing Unicef framework of malnutrition by adding enteropathogen infection in the absence of diarrhoea.

## Introduction

Growth faltering during early childhood is associated with increased risk of illness and death^1 2^ and, over the long term, fewer years of schooling and lower earning potential.^3^ Undernutrition arises from diet inadequacies and high illness burden, particularly due to diarrhoea, which in turn arise from socioenvironmental factors characterising poverty.^2 4^ The negative impact of diarrhoeal illness on growth is well established,^5^ with some diarrhoeal pathogens having a more severe impact on growth than others.^6 7^ Although less established, elevated exposure to enteropathogens in apparently healthy children has also been associated with growth faltering.^8 9^ Even in the absence of diarrhoeal illness, enteric pathogens are thought to contribute to undernutrition and growth faltering by impairing gut function, altering nutrient uptake and metabolism and modulating immunity.^10 11^

The Etiology, Risk Factors and Interactions of Enteric Infections and Malnutrition and the Consequences for Child Health and Development (MAL-ED) study is a longitudinal cohort study of children enrolled shortly after birth and followed until 24 months of age in eight sites with historically high prevalence of undernutrition.^12^ A key goal of the MAL-ED study is to assess the role of enteropathogens and other factors in growth faltering across the sites. Here, we assess whether enteropathogen burden, diarrhoeal morbidity and variation in breastfeeding practices and complementary food intakes affect rates of growth in early childhood, including the potential for catch-up growth, as well as attained length and weight at 24 months.

## Methods

### Study design and population

The MAL-ED study was conducted from November 2009 to February 2014 in Bangladesh (Dhaka (BGD)), India (Vellore (INV)), Nepal (Bhaktapur (NEB)), Pakistan (Naushehro Feroze (PKN)), Brazil (Fortaleza (BRF)), Peru (Loreto (PEL)), South Africa (Venda (SAV)) and Tanzania (Haydom (TZH)).^12^ Each site obtained ethical approval from their respective institutions and written informed consent from participants. Newborns were enrolled within 17 days after birth. Exclusion criteria were: very low birth weight (<1500 g), very ill, non-singleton and mother less than 16 years old. Cohort enrolment was staggered (~10 per month), with the goal of retaining at least 200 per site at 24 months of age. The study design is described elsewhere,^12^ as are data collection methods for illness and treatment,^13^ infant feeding^14^ and stool microbiology.^15^

#### Length and weight

The primary outcome measures were length (cm) and weight (kg), which were measured at enrolment and monthly thereafter by trained personnel following standard procedures.^16^ To ensure quality control, extreme measurements were investigated and secondary measurements were collected within 24 hours for ~5% of all metrics. Reliability estimates (R) for both weights and lengths were >0.9. Bias was identified in a subset of PKN length measurements; therefore, PKN data have been excluded from these analyses. To capture changing growth rates in early childhood, we delineated six age-based time periods: enrolment to 2 months, 3–5 months, 6–8 months, 9–11 months, 12–17 months and 18–24 months. Growth in length or weight during each of those periods was modelled using monthly anthropometry measures as a function of the exposure variables described below. To describe the nutritional status of the populations, we converted lengths and weights to Z-scores.^17^

#### Twice-weekly visits

Illness symptoms and infant feeding data were collected during twice-weekly visits to the household. More than 80% of children were visited twice a week during each time period, and <20% of children had a gap in follow-up longer than 1 week during the first 2 years of life. On average, children at the sites were visited 200 times during the 2-year period (mean number of visits at the sites ranged from 189 to 207).

#### Illness

During twice weekly home visits, families were asked about the symptoms children had experienced since the last visit (eg, loose and/or bloody stools, fever, vomiting, and cough, among others) and any treatments they received.^13^ Diarrhoeal illness was defined as ≥3 loose stools in a 24-hour period, or at least one loose stool with blood, and episodes were separated by at least two diarrhoea-free days.^18 19^ We considered alternative characterisations of diarrhoea (eg, severe, persistent, prevalence and incidence), as well as other illnesses (eg, respiratory infections, fever and cough), for inclusion in the models and ultimately chose to include only the number of untreated (by antibiotics) episodes of diarrhoea during each of the age periods for each child based on improvement in the Akaike Information Criterion (AIC).

#### Diet

Infant feeding practices over time were characterised by collecting and integrating several variables.^14^ During twice-weekly surveillance visits, feeding practices were collected for the previous day. Breastfeeding data were then characterised as full (exclusive or predominant, ie, received only breast milk or breast milk with water or clear liquids), partial (received breast milk plus other milk or food) or none.^20^ Information about the feeding patterns was carried forward until the next available surveillance visit data. Greater detail regarding dietary intakes was collected monthly, and those monthly data were also incorporated into the breastfeeding categorisation. From 1 to 8 months, trained staff inquired about the intake of specific food groups on the previous day. To evaluate changes in feeding patterns, children were categorised as being fully breast fed >95% of days at 0–2 months (yes/no), fully breast fed or partially breast fed with the addition of dairy >95% days at 3–5 months (yes/no) and fed any dairy products >95% days at 6–8 months (yes/no). Other aspects of early childhood feeding were considered (eg, feeding frequency and dietary diversity), and we chose these based on variability within and among the sites and on improvement in AIC.

Monthly from 9 to 24 months, we collected 24 hour recalls, and those recalls were linked to site-specific food composition databases created within MAL-ED to quantify energy, macronutrient and micronutrient intakes from non-breast milk foods. We calculated the mean energy and protein intakes at 9–11, 12–17 and 18–24 months for each child. We regressed protein intake data against the energy intake using linear mixed effects regression (separate models for each site with random intercepts per child) and retained the residuals (which represent energy-adjusted protein intake) for analysis.^21^ Initial analyses revealed relationships between protein residual and growth, and this led us to consider related dietary variables (eg, zinc intake and protein from animal source foods) for inclusion in the model, but these were not retained based on fit.

#### Microbiology

Surveillance stool samples were analysed monthly through the first year and quarterly thereafter.^15^ Each sample was tested for an extensive panel of bacterial, viral and parasitic enteropathogens, and we included in the analysis only those samples that were tested for the full complement of (40+) assays. To avoid overlap with diarrhoeal illness, which was examined separately, stool samples collected during or within 2 days of a diarrhoeal episode were excluded from the analyses. For each time period, enteropathogen detections were calculated as the number of pathogens detected divided by the number of samples for each child.

Additionally, the top six pathogens by prevalence^22^ in the surveillance stool samples (*Campylobacter* and enteroaggregative *Escherichia coli* (EAEC), atypical enteropathogenic *E. coli* (EPEC), *Giardia*, heat-labile toxin producing enterotoxigenic *E. coli* (LT-ETEC) and *Cryptosporidium*) were examined separately to evaluate their individual effects on growth. These were included in the model as present or absent for each child in each time period. Only *Campylobacter*, EAEC and *Giardia* were retained in the final model based on the AIC.

#### Socioeconomic status

Caregivers were queried about their income, assets, education, type of sanitation and source of drinking water every 6 months, beginning at 6 months of age. These variables were combined to form a Water, Assets, Maternal education and household Income (WAMI) index.^23^ In order to remove the potential effect of seasonality on the WAMI index, we took the mean WAMI values, by child, from surveys conducted over the study.

### Statistical analysis

A linear piecewise regression spline model that included all monthly anthropometry data with six time periods (0–2, 3–5, 6–8, 9–11, 12–17 and 18–24 months) was used to evaluate the effects of time-varying influences on growth trajectories.^24 25^ The linear spline method was chosen because children grow at different rates over time (higher rates of growth in early childhood which decrease during the first 2 years of life) and children are exposed to different feeding practices and infections at different ages. The knots allow each segment to have a different slope (growth rate) that can vary based on different exposures. The selection of variables included in the model was based on prior evidence of a relationship with growth and three criteria: (1) clear biological rationale; (2) reduction in the random effects between children (ie, maximising the explanatory power of a fixed effect); and (3) improvement in model fit (based on AIC). The Statistical Analysis System (SAS) code for the model is provided in online supplementary table 1, and supplementary table 2 provides a list of variables considered for inclusion in the models.

10.1136/bmjgh-2017-000370.supp1 Supplemental materials 1

The model included child-level random effects on the intercept and first age (0–2 months) slope to account for the repeated measures of individual children and allow for child-specific growth trajectories. Additional random effects were not supported by the data. To account for within-child serial correlation, a first-order continuous autoregressive error was incorporated. Also included were study site, sex, length-for-age and weight-for-age at enrolment and WAMI. Interactions between terms and each piecewise age segment were included based on improvements in the AIC (ΔAIC >2). We examined the inclusion of all diarrhoeal episodes, severe diarrhoea, and persistent diarrhoea, as well as diarrhoeal episodes that were or were not coincident with antibiotic use (34 466/88 668 (39%); 54 202/88 668 (61%), respectively) to address confounding of care-seeking, the role of antibiotics and the distinction between symptoms and infection. Only diarrhoeal episodes not coincident with antibiotics were associated with growth rates and therefore included in the final model. We evaluated total pathogen burden, and in a second model, evaluated the six most common pathogens. The pathogen detection, diarrhoea and complementary feeding variables each had lagged effects (by one period) that we included to evaluate whether factors affecting growth in one period continued to be associated with growth rates in the next period. All continuous variables were centred at each time period.

Using linear combinations of model parameters, we estimated the cumulative impacts of pathogen detections, diarrhoeal illness and diet on length and weight at 24 months. Predictions of length and weight at 24 months were generated for mean, low (10th percentile) and high (90th percentile) enteropathogen, diarrhoea and dietary exposures (estimated for each period) (numeric results are presented in online supplementary tables 3 and 4. Site-specific models were run using 10th and 90th percentiles for risk factors that were specific to the site. For individual pathogens, we estimated the effect of any positive test for that pathogen in each period versus no positive test. The difference between the mean exposures and the low or high scenarios are expressed as length or weight (in cm or kg and in Z-scores) estimated at 24 months assuming mean values for all other continuous variables (eg, WAMI) and 0 for all binary variables (eg, <95% days of full breast feeding in the 0–2 months age period).

Of the 1868 children enrolled in seven sites included in these analyses, 11 died, 269 moved away and 87 dropped out or their status was unknown. Among the 1501 children remaining, three did not have at least one anthropometric measurement in each period, 180 children did not have at least one non-diarrhoea stool sample in each period and 27 children did not have at least one 24-hour dietary recall in each period, leaving 1291 (69%) children who met the minimum standard and were retained (table 1). Analyses comparing characteristics of those included/excluded from analyses revealed small differences in characteristics within individual sites but no consistent differences across sites (online supplementary table 5). All 86 children lost due to missing pathogen history in BRF did not have any fully tested stool samples in the first 3 months of life.

**Table 1:** Subjects included in the analysis, from enrolment (top row) to final number with complete data (bottom row), N (%)

	Southern Asia			Latin America		Sub-Saharan Africa		
	BGD	INV	NEB	BRF	PEL	SAV	TZH	Total
Enrolled	265	251	240	233	303	314	262	1868
Complete anthropometry*	213 (80)	228 (91)	228 (95)	169 (73)	208 (69)	237 (75)	215 (82)	1498 (80)
Complete anthropometry* and pathogen history†	206 (78)	200 (80)	208 (87)	83 (36)	198 (65)	217 (69)	206 (79)	1318 (71)
Complete anthropometry*, pathogen history† and complementary diet data‡	206 (78)	200 (80)	208 (87)	81 (35)	198 (65)	199 (63)	199 (76)	1291 (69)

*Children with baseline anthropometry, at least one weight and length measurement in each time period (0–2, 3–5, 6–8, 9–11, 12–17 and 18–24 months), and last anthropometry at 22+ months. Lost in this stage are 11 children who died, 269 children who moved away and 87 children who dropped out or their status was unknown, as well as three children who did not have at least one anthropometry measurement in each period.

†Children with at least one fully tested surveillance stool in each time period (0–2, 3–5, 6–8, 9–11, 12–17 and 18–24 months); 591 children (46%) had only one fully tested surveillance stool during any time period.

‡Children with at least one quantitative complementary diet recall in each time period (9–11, 12–17 and 18–24 months); 87 children (6.7%) had only one 24-hour recall during any time period.

Sites: BGD, Bangladesh (Dhaka); INV, India (Vellore); NEB, Nepal (Bhaktapur); BRF, Brazil (Fortaleza); PEL, Peru (Loreto); SAV, South Africa (Venda); TZH, Tanzania (Haydom).

Data within each period were generally complete. For example, more than 94% of the children had complete anthropometric records in each period (three during the first four periods and six in the last two periods). The completeness of the stool samples was lower than that for anthropometry due to the difficulty in obtaining analysable samples (eg, in a timely fashion and of sufficient quantity) and the requirement for complete microbiological testing of those samples. On average, we collected and tested 14 non-diarrhoea stool samples per child over the first 2 years of life (range of the mean number of samples per child at the sites was 13–15). Similarly, on average, 15 dietary recalls between 9 and 24 months were used to estimate usual nutrient intakes.

Statistical analyses were performed using SAS V.9.3, and figures were generated using R V.3.2.2 (Foundation for Statistical Computing, Vienna, Austria).

## Results

### Characteristics of the cohort

At enrolment, mean LAZ ranged from −1.0 to −0.7 and mean WAZ ranged from −1.3 to −0.1 across the sites (table 2). By 24 months, children in BRF achieved the greatest mean lengths and weights (LAZ 0.0, WAZ 0.3), in contrast to the shortest mean length in TZH (−2.7 LAZ) and lowest mean weight in INV (−1.6 WAZ). Overall, 524/1291 (41%) of the children were stunted (LAZ <−2) (range 3/81 (3.7%) in BRF to 142/199 (71.4%) in TZH) and 243/1291 (19%) were underweight (WAZ <−2) (range 2/81 (2.5%) in BRF to 70/200 (35%) in INV) at 24 months of age.

**Table 2:** Selected characteristics (% or mean (range)) of the children included in the analysis

	Southern Asia			Latin America		Sub-Saharan Africa	
	BGD	INV	NEB	BRF	PEL	SAV	TZH
N	206	200	208	81	198	199	199
Age at enrolment (days)	3 (0,15)	10 (1,17)	11 (3,17)	9 (1,16)	5 (1,17)	9 (1,17)	7 (1,17)
Males (%)	51	44	54	64	57	51	50
Enrolment LAZ	−1.0 (−4.1, 1.0)	−1.0 (−5.0, 1.5)	−0.7 (−3.9, 1.3)	−0.8 (−5.0, 1.8)	−0.9 (−4.0, 2.0)	−0.7 (−4.0, 2.0)	−1.0 (−4.3, 2.1)
Enrolment WAZ	−1.3 (−4.0, 0.8)	−1.3 (−4.8, 1.3)	−0.9 (−4.1, 1.4)	−0.1 (−3.6, 2.5)	−0.6 (−3.8, 2.1)	−0.4 (−3.2, 1.6)	−0.1 (−3.3, 2.1)
Enrolment WLZ	−1.0 (−4.1, 1.7)	−1.2 (−5.2, 1.6)	−0.9 (−5.5, 2.3)	0.6 (−2.6, 4.1)	−0.05 (−2.7, 3.3)	−0.01 (−4.8, 2.8)	0.7 (−3.1, 3.9)
Income (US$)	130 (36, 924)	73 (9, 220)	189 (14, 932)	364 (0, 901)	137 (4, 429)	280 (34, 2208)	29 (1, 240)
Protected water source (%)	100	100	100	100	96	83	42
Latrine or toilet (%)	100	51	100	100	32	98	10
WAMI score	0.5 (0.3, 0.9)	0.4 (0.2, 0.9)	0.7 (0.4, 1)	0.8 (0.5, 0.9)	0.5 (0.2, 0.9)	0.8 (0.3, 1)	0.2 (0, 0.6)

BGD, Bangladesh (Dhaka); BRF, Brazil (Fortaleza); INV, India (Vellore); NEB, Nepal (Bhaktapur); PEL, Peru (Loreto); SAV, South Africa (Venda); TZH, Tanzania (Haydom); LAZ, length-for-age z-score; WAZ, weight-for-age z-score; WLZ, weight-for-length z-score; WAMI, Water, Assets, Maternal education and household Income.

As children aged, pathogen detections in the absence of diarrhoea increased from a mean of 0.5 pathogens/stool at 0–2 months to 1.3 pathogens/stool at 18–24 months (table 3). *Campylobacter* was identified in around half the cohort at least once in each period from 6 months onwards; the same was true for EAEC from 3 months onwards. Children had a mean 0.2–0.4 diarrhoeal episodes, on average, that were not coincident with antibiotic use in each of the 3-month periods during the first year and a mean 0.5–0.7 episodes for each 6-month period in the second year.

**Table 3:** Key dietary, asymptomatic pathogen and illness exposures by age-based time period

Variable	Time period					
	**0–2** months	**3–5** months	**6–8** months	**9–11** months	**12–17** months	**18–24** months
Growth velocity*						
Length (cm/month)	3.8 (2.6, 4.9)	2.1 (1.3, 3.0)	1.3 (0.6, 2.1)	1.0 (0.4, 1.8)	0.9 (0.6, 1.2)	0.8 (0.5, 1.1)
Weight (kg/month)	1.1 (0.7, 1.5)	0.5 (0.3, 0.8)	0.3 (0.1, 0.5)	0.2 (0, 0.4)	0.2 (0, 0.3)	0.2 (0.1, 0.3)
**Breast feeding**						
Exclusive (percent of days)	66 (17, 100)	28 (0, 85)	2 (0, 4)	<1 (0, 0)	<1 (0, 0)	<1 (0, 0)
Predominant (percent of days)	12 (0, 44)	12 (0, 42)	5 (0, 16)	2 (0, 8)	1 (0, 4)	<1 (0, 0)
Any (percent of days)	99 (100, 100)	97 (100, 100)	95 (100, 100)	92 (92, 100)	81 (4, 100)	53 (0, 100)
**Complementary food**						
Dairy (percent of days)	15 (0, 60)	33 (0, 100)	47 (0, 100)	52 (2, 100)	57 (8, 100)	62 (11, 100)
Energy intake (kcal/day)				482 (140, 952)	628 (239, 1059)	865 (407, 1284)
Protein (g/day)				15 (4, 31)	19 (6, 35)	26 (11, 42)
**Illness**						
Antibiotic use (days in period)	4 (0, 14)	6 (0, 18)	7 (0, 18)	7 (0, 18)	12 (0, 30)	10 (0, 26)
Diarrhoea† (episodes in period)	0.2 (0, 1)	0.4 (0, 1)	0.4 (0, 1)	0.4 (0, 1)	0.7 (0, 2)	0.5 (0, 2)
**Pathogens in non-diarrhoeal stool**						
Mean detections/stool	0.5 (0, 1.0)	0.7 (0, 1.5)	1.0 (0, 2.0)	1.2 (0.3, 2.0)	1.3 (0.5, 2.5)	1.3 (0.3, 2.5)
*Campylobacter* (% with any detection in period)	12	30	48	59	49	59
EAEC (% with any detection in period)	26	51	60	60	47	48
*Giardia* (% with any detection in period)	1	3	9	16	25	48
Atypical EPEC (% with any detection in period)	3	10	16	21	13	16
LT-ETEC (% with any detection in period)	4	9	12	16	12	16
*Cryptosporidium* (% with any detection in period)	7	7	7	10	9	10

Mean (10th, 90th percentiles).

*Velocities presented in this table are calculated as the difference between the last and first measurements within each period divided by the number of months in same period for each child and then taking the average for the cohort.

†Diarrhoea episodes that were not coincident with antibiotic use.

EAEC, enteroaggregative *Escherichia coli*; EPEC, enteropathogenic *Escherichia coli*; LT-ETEC, LT-producing enterotoxigenic *Escherichia coli*.

Most children were breast fed at birth. Children at the sites were, on average, exclusively breast fed for the first 2 months of life. Exclusive breast feeding declined by half in the period of 3–5 months. Although exclusive breast feeding was not common beyond 3 months of age at most sites, breast milk continued to be part of the diet on around half of all days in the period of 18–24 months across the study population. The inclusion of animal milks and/or dairy was notable at 3–5 and 6–8 months. From 9 to 24 months, there was considerable variation in the energy and protein intakes from non-breast milk foods among children.

### Relationships with length and weight

With respect to time invariant factors, higher socioeconomic status (WAMI) was associated with greater linear and ponderal growth velocity (0.018±0.003 cm increase in length per month for each 10% increase in WAMI (p<0.001); 0.007±0.001 kg increase in weight per month (p<0.001)). Higher enrolment LAZ was associated with lower linear growth velocity in the first two age periods (0–2 months, −0.23±0.02 cm per month (p<0.001); 3–5 months, −0.12±0.02 cm per month (p<0.001)) and had no statistically significant relationship with growth velocity thereafter. Similarly, higher enrolment WAZ was associated with lower ponderal growth velocity in the first two age periods (0–2 months, −0.02±0.005 kg per month (p=0.007); 3–5 months, −0.01±0.005 kg per month (p=0.02)). Child sex influenced linear growth velocities during early childhood (boys grew 0.26±0.04 cm faster per month (p<0.001) than girls in the first 3 months, 0.14±0.02 cm faster per month (p=0.02) in the 3–5 month period and 0.03±0.007 cm slower per month (p<0.001) thereafter), but sex did not significantly modify the effect of the time-varying covariates. Child sex had a similar relationship with weight velocity.

**Figure 1:**
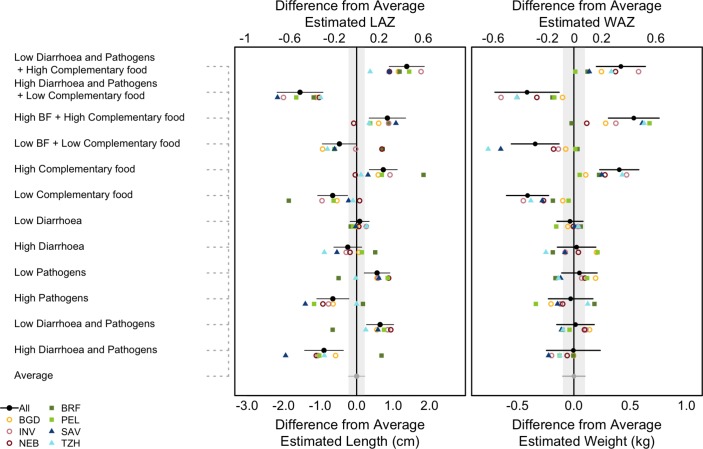
(Left) The predicted difference in length (bottom axis) and length-for-age z-score (LAZ) (top axis) and (right) weight (bottom axis) and weight-for-age z-score (WAZ) (top axis) at 24 months between scenarios that alter potential risk factors and a scenario based on the average experience of children in the cohort. Absolute length and weight were converted into Z-scores using the WHO Growth Standards.^17^ The high and low scenarios are based on changing the named variable(s) to the 90th and 10th percentiles of their observed distribution respectively while holding all other variables at their mean level. BF refers to % days of full breast feeding from 0 to 5 months, and complementary food refers to the % of days fed animal milks and dairy between 3 and 8 months, and energy and (energy-adjusted) protein intakes from non-breast milk foods from 9 to 24 months. Horizontal lines indicate the 95% CI around the mean differences. The grey vertical bars indicate the 95% CI around the average estimate (which has 0 difference from itself). Site-specific models used 10th and 90th percentiles for risk factors that were specific to the site. Sites: BGD, Bangladesh (Dhaka); INV, India (Vellore); NEB, Nepal (Bhaktapur); BRF, Brazil (Fortaleza); PEL, Peru (Loreto); SAV, South Africa (Venda); TZH, Tanzania (Haydom).

Higher rates of enteropathogen detections in non-diarrhoeal stools were associated with slower linear growth. Enteropathogen detections were less frequent in early childhood, but the relationship with growth rates in the first 3 months of life was negative and not fully recovered in the following period (0–2 months, −0.07±0.02 cm per month (p=0.004); 3–5 month rebound, 0.06±0.02 cm per month (p=0.02)). Enteropathogen detections were often associated with slower growth within a particular period and with rebound in the following period; however, full recovery was not attained, leading to lower mean attained length at 24 months. As shown in figure 1, children with high enteropathogen detections in non-diarrhoeal stools were estimated to be 1.21±0.33 cm (p<0.001, equivalent to 0.39 LAZ) shorter at 24 months than children with low burdens.

The relationship between enteropathogens and weight was less consistent, and no statistically significant relationship overall was observed at 24 months. The association between pathogens and weight was most notable in the first 3 months of life (−0.04±0.01 kg per month (p<0.0001)) but was fully recovered in the following period (0.04±0.01 kg per month (p<0.0001)). Full recovery in weight was consistent in all age periods. Based on the model, children with high enteropathogen detections in non-diarrhoea stools were not found to weigh significantly less than children with low enteropathogen detections (high enteropathogen detections: 0.08±0.15 kg lighter at 24 months (p=0.60)) (figure 1).

The contribution of diarrhoeal morbidity was small relative to that of enteropathogen exposure in the absence of diarrhoeal symptoms. The short-term association between diarrhoeal morbidity and linear growth was consistently negative (between −0.02 and −0.03 cm per month in each time period after 3 months of age), but the growth predicted in the following period consistently offset the short-term faltering, leading to no statistically significant long-term association between diarrhoea and linear growth (children in the 90th percentile for diarrhoea episodes were 0.33±0.22 cm shorter (p=0.13) than children in the 10th percentile for diarrhoea) (figure 1). The association between diarrhoeal morbidity and weight was smaller and less consistent (children with a high diarrhoea burden were 0.06±0.1 kg heavier than children with a low diarrhoea burden (p=0.55)).

**Figure 2:**
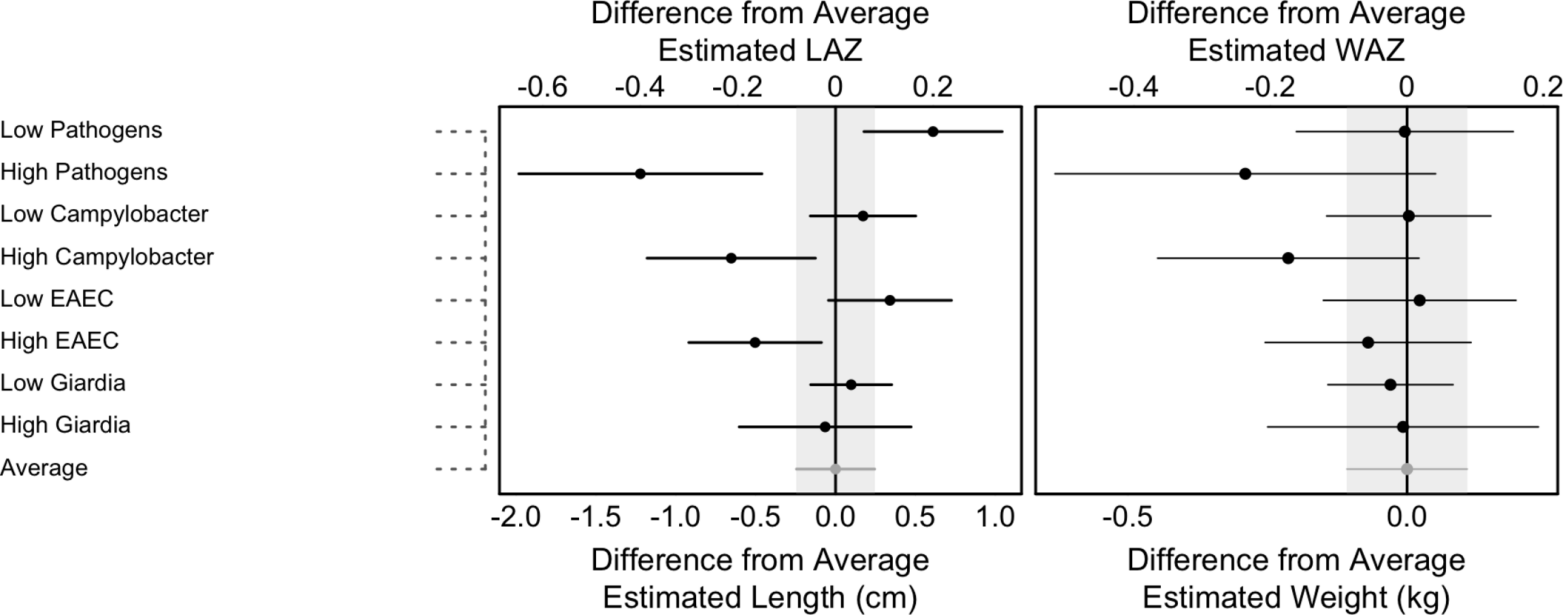
The predicted difference in length (bottom axis) and LAZ (top axis) (left) and weight (bottom axis) and WAZ (top axis) (right) at 24 months based on scenarios that change individual factors potentially affecting growth velocity adjusting for the mean of all factors. The absolute length and weight were converted to z-scores for length-for age (LAZ) and weight-for-age (WAZ) using the WHO Growth Standards.^17^ The high and low scenarios are based on presence or absence of the named pathogen in at least one surveillance stool in each period while holding all other variables at their mean level. The pathogens represented here are the top three pathogens by prevalence (*Campylobacter*, EAEC and *Giardia*). Horizontal lines indicate the 95% CI around the mean differences. The grey vertical bars indicate the 95% CI around the average estimate (which has 0 difference from itself). Sites: BGD, Bangladesh (Dhaka); INV, India (Vellore); NEB, Nepal (Bhaktapur); BRF, Brazil (Fortaleza) PEL, Peru (Loreto); SAV, South Africa (Venda); TZH, Tanzania (Haydom). EAEC, enteroaggregative *Escherichia coli.*

Among the six pathogens evaluated for their specific effects on growth, *Campylobacter* was associated with lower growth rates for both length and weight. At 24 months, children with *Campylobacter* detected in every period were predicted to be 0.83±0.33 cm shorter (p=0.01) and 0.22±0.15 kg lighter (p=0.14) than children with no exposure (equivalent to a mean difference of 0.27 LAZ and 0.22 WAZ) (figure 2). Similarly, children with detections of EAEC in each period were, on average, 0.85±0.31 cm shorter (p=0.001) and 0.09±0.14 kg lighter (p=0.52) than children with no detections (0.28 LAZ, 0.09 WAZ). *Campylobacter* additionally showed an age-related pattern such that the mean effect of detections was greater at 0–2 months and decreased thereafter. Though tending towards lower growth velocities, no consistent, long-term relationship between *Giardia*, *Cryptosporidium*, LT-ETEC or atypical EPEC and growth were found, perhaps due to lower prevalence of these infections leading to limited power to detect a difference.

With respect to dietary intake, better breastfeeding practices were not associated with higher linear growth velocity at 0–2 and 3–5 months (full breast feeding in 0–2 months, 0.01±0.03 cm per month (p=0.62); fully breast fed or partially breast fed with the addition of dairy in 3–5 months, 0.02±0.02 cm per month (p=0.38)). Inclusion of animal milks and/or dairy at 6–8 months was associated with a small but significantly higher linear growth velocity (0.08±0.03 cm per month (p=0.004)). Intakes of energy and energy-adjusted protein (protein content) from non-breast milk foods from 9 to 24 months were associated with higher child growth velocity and influenced attained size at 24 months (figure 1). At 24 months, children with lower complementary food intakes were, on average, 1.39±0.33 cm (p<0.001, 0.46 LAZ) shorter and weighed 0.81±0.15 kg (p<0.001, 0.65 WAZ) less than children with higher intakes from complementary foods. We found detectable but weaker associations for energy-adjusted zinc and vitamin B_12_ intakes, suggesting that a higher protein content diet was associated with the provision of animal source foods, with milk and meat identified as important contributors to greater protein density across the sites.

## Discussion

The relationship between diarrhoeal morbidity, dietary intakes and growth during early childhood within the context of poverty has been the subject of research since the 1950s and 1960s.^5 26^ The majority of studies contributing to estimated impacts of diarrhoea frequency and severity and poor infant feeding practices on undernutrition and mortality were conducted in the 1970s–1990s.^2^ This research led to initiatives to reduce morbidity and improve dietary intakes of children, particularly during illness. Here, we demonstrate the association of an extensive panel of bacterial, viral and parasitic enteropathogens with the growth deficits experienced in children from 0 to 24 months in diverse settings over three continents. This result is unique both because it tracks longitudinal cumulative insults on growth and because it provides support for the debilitating effect of enteropathogens distinct from overt illness. Furthermore, we have demonstrated that *Campylobacter* and EAEC were frequently detected in the absence of diarrhoea^22^ and that each was associated with accumulated growth deficits by 24 months. These specific pathogens have been examined in greater detail elsewhere.^27 28^

The relationship between diarrhoeal morbidity and growth was small relative to that of enteropathogen presence, regardless of whether all diarrhoea episodes were examined or limited to those not coincident with antibiotic use. This limited association was surprising. One possible explanation is that the incidence, prevalence and severity of diarrhoea found in this study^22^ were diminished compared with studies from prior decades, perhaps because many families participating in the MAL-ED study had access to improved water sources and a latrine or toilet.^23^ Although this was an observational study that provided no treatment for illness, study personnel referred children to the healthcare system and families often sought treatment, including antibiotics. Thus, it may not be surprising that an association between diarrhoeal morbidity and growth over time is detectable but relatively small and limited to only those episodes that were not coincident with antibiotic treatment (ie, episodes that likely followed a natural course and were not confounded by care-seeking).

Variation in breastfeeding practices was not associated with growth in any age period but, as noted, the vast majority of children were breast fed, and the durations of exclusive breast feeding were relatively short in most sites.^29^ Predominant and/or partial breast feeding has been shown to increase risk of diarrhoeal illness in infants compared with exclusive breast feeding,^2^ but as shown here, these infants experience relatively few episodes of diarrhoea, and many episodes are associated with use of antibiotics.

Our results indicate that improving the quantity and quality of intakes from complementary foods from 9 to 24 months would improve growth rates and attained length and weight at 24 months. Because most children were breast fed over the entire period, their total energy and protein intakes are difficult to evaluate. However, study children may have received less energy and nutrients from breast milk than expected compared with values reported in the literature,^30^ or they may have had higher requirements because of illness, inflammation or catch-up growth.^31^ Across sites, higher energy-adjusted protein intakes were achieved through greater consumption of animal-source foods (eg, milk and meat) that could indicate either greater overall nutrient density or exposure to factors that stimulate growth.^32^ Reanalyses of children from the Guatemalan and Cebu cohort studies has also demonstrated the importance of the protein density of complementary foods for growth.^33^

Importantly, children may experience multiple negative exposures over time (eg, simultaneous enteropathogen infection, untreated morbidity and low complementary food intake). Summing the effects shown in figure 1, these combined effects lead to growth deficits on the order of 2.93±0.51 cm (p<0.001, 0.98 LAZ) and 0.83±0.23 kg (p<0.001, 0.73 WAZ) for length and weight, respectively, comparing the best and worst case scenarios. The deficits accumulate because, although the effect of each insult may be transient, recovery (particularly for linear growth) may be incomplete when children suffer consecutive insults.

When fit to each site, our model demonstrated greater consistency among the findings related to diet than for pathogens, likely due to greater heterogeneity in pathogen prevalence between sites. This variation was even more pronounced for individual pathogens. Additionally, although we adjusted for both within-child clustering (as a random effect) and for site (as a fixed effect), as well as socioeconomic status, there was still substantial unexplained variation in growth both within and between sites. Further analyses of these data may reveal additional factors affecting growth at individual sites.

A central hypothesis of the MAL-ED study is that repeated enteric infections lead to growth faltering through impaired gut permeability and inflammation. In this work, we show that the first part of this hypothesis is true. In other work,^34^ we evaluated relationships between enteropathogen presence, biomarkers of gut permeability and inflammation and growth velocity. While some of the pathways evaluated in that paper have statistical support, particularly for enteropathogens such as *Campylobacter* and EAEC, the overall strength of associations does not implicate gut permeability and inflammation (as measured) as a clear explanatory factor for how cumulative enteropathogen exposure leads to growth faltering.

Strengths of this study included the unequalled intensity of enteropathogen testing of this study (40+ pathogens in 18 430 non-diarrhoeal stools from 1291 children). Few children were lost from this study and, of those who remained, 69% had complete data for these analyses. The harmonised protocol, well-trained personnel and multiple quality assurance measures ensured the reliability of key measures. In addition, we controlled for some of the major potential confounding factors, socioeconomic status (which included aspects of water and sanitation, maternal education and income) and size at birth in the model. Despite these strengths, some limitations are recognised. This is a longitudinal cohort, and we therefore cannot assess factors that were relatively homogenous at the sites, nor can we directly ascertain causality. Removing PKN anthropometric data was unfortunate and negatively impacted our sample size. Also, although we found little evidence of selection bias, completeness of pathogen testing from BRF was low in the first several months of life and resulted in a large number of children who were ineligible for the analysis. Additionally, we were not able to fully evaluate specific contributions of less common enteropathogens, nor can one easily differentiate new infections from persistent carriage to compare consequences for growth. Finally, because we could not characterise breast milk intakes, we were unable to evaluate the impact of total diet on child growth.

Even with these limitations, the results of this study identify asymptomatic enteropathogen burden as an important contributor to growth faltering in children, distinct from diarrhoeal illness. Interventions are needed to reduce exposure to enteropathogens, as well as diarrhoeal illness, in young children, and specifically to reduce infections with *Campylobacter* and EAEC based on their high frequency of detection and negative impact on growth. Concurrently, improvements in the quantity and (protein) quality of complementary food intakes could reduce growth faltering, underscoring the need for expanded and sustained action to improve young child feeding in the context of poverty.
