# Supplementary material for: Relationship between growth and illness, enteropathogens and dietary intakes in the first 2 years of life: findings from the MAL-ED birth cohort study
Source: BMJ Glob Health. 2017 Dec 28;2(4):e000370. doi: 10.1136/bmjgh-2017-000370 (PMC5759708; doi:10.1136/bmjgh-2017-000370)
Supplement: Supplemental materials 1 [file bmjgh-2017-000370supp001.pdf]

Online Supplemental Materials

Supplemental Table 1. SAS code used to generate the model. This code is provided to demonstrate the logic of the model and support reproducible methods.

SAS Command:

```
proc mixed data=gf.allnoPK  ;
class pid country_id;
model length= laze_c waze_c ma0fe1 country_id age age3 age6 age9 age12 age18
        laze_c*age laze_c*age3 laze_c*age6
        ma0fe1*age ma0fe1*age3 ma0fe1*age6
        country_id*age country_id*age3 country_id*age6 country_id*age9
country_id*age12 country_id*age18
        meanwami_c meanwami_c*age
        epbfpb950*age eppdpb951*age3 dairy952*age6
        epbfpb950*age3 eppdpb951*age6 dairy952*age9
        sumkcal_s_c3*age9 sumkcal_s_c4*age12 sumkcal_s_c5*age18
        sumkcal_s_c3*age12 sumkcal_s_c4*age18
        procal3*age9 procal4*age12 procal5*age18
        procal3*age12 procal4*age18
        dint3nab_c0*age dint3nab_c1*age3 dint3nab_c2*age6 dint3nab_c3*age9
dint3nab_c4*age12 dint3nab_c5*age18
        dint3nab_c0*age3 dint3nab_c1*age6 dint3nab_c2*age9 dint3nab_c3*age12
dint3nab_c4*age18
        dens_c0*age dens_c1*age3 dens_c2*age6 dens_c3*age9 dens_c4*age12
dens_c5*age18
        dens_c0*age3 dens_c1*age6 dens_c2*age9 dens_c3*age12 dens_c4*age18
/solution outp=outp outpm=outpm;
random intercept age /subject=pid solution;
repeated /type=sp(pow)(age) sub=pid;
```

Key:

pid	child identifier
country_id	site identifier
length	dependent variable, either length (cm) (as shown) or weight (kg)
laze_c	length-for-age z-score at enrolment
waze_c	weight-for-age z-score at enrolment
ma0fe1	child sex (coded as 0, males; 1, females)
age age3 age6 age9 age12 age18	age in terms of spline knots (at months 3, 6, 9, 12, 18)
meanwami_c	mean average WAMI index as a measure of SES
epbfpb950 eppdpb951	proportion of days with either exclusive or predominant breast feeding in the 0-2m age or 3-6m age periods respectively

dairy952	dairy intake in the 6-9m period
sumkcal_s_c3 sumkcal_s_c4 sumkcal_s_c5	Total energy intake during the 9-11, 12-17 and 18-24m period respectively
procal3 procal4 procal5	protein intake adjusted for energy intake during the 9-11, 12-17 and 18-24m period respectively
dint3nab_c0 dint3nab_c1 dint3nab_c2 dint3nab_c3 dint3nab_c4 dint3nab_c5	diarrhoea episodes, not coincident with antibiotic use, in each of the age periods
dens_c0 dens_c1 dens_c2 dens_c3 dens_c4 dens_c5	the number of pathogens detected/ number of stools assayed in each of the age periods

Note 1, the suffix _c is used to indicate variables that have been centered, i.e. scaled to have mean 0

Note 2, the suffixes 0-5 are used to indicate the 6 linear piecewise age segments (0, 0-2m; 1, 3-5m; 2, 6-8m; 3, 9-11m; 4, 12-17m; 5, 18-24m)

Supplemental Table 2. Candidate variables for the linear spline models of weight and length. Interaction with age identifies the age period in the spline model

Variables	Format	Retained	Interaction with age	Detailed description	Support for being included or excluded from final model
**Overall and baseline characteristics**					
WAMI	Mean score	YES	1	The wami is calculated by summing values of the following variables: sanitation, asset, incat, newfseschool, and dividing the total by 32	Incorporates aspects of maternal education, income and water and sanitation, and smooths out differences among sites
Maternal education	Years			Mother's education	Included in WAMI, therefore redundant
Improved sanitation	Yes 1, no 0			Score of sanitation at baseline; 0 for unimproved categories, 1 for improved categories : if fsetoiletmod = No facility/bush/field or bucket toilet/pit latrine without slab then unimproved; if fsetoiletmod = Pit latrine without flush/with slab(improved), Flush to piped sewer system, Flush to septic tank, Flush to pit latrine, Flush to somewhere else, Other = 07, then improved	Included in WAMI, therefore redundant
Improved drinking water	Yes 1, no 0			if fsewdrink = Piped into dwelling, Piped to yard/plot, Public tap/stand pipe, Tube well or borehole, Protected well, or Other then improved. If fsewdrink = Unprotected well, Surface water (river/ dam/ lake/pond/ stream/canal/irrigation canal), then unimproved. Exception: PEL where fsewdrink=Other is also considered	Included in WAMI, therefore redundant

				unimproved due to site-specific circumstances	
Any food insecurity	Yes 1, no 0			FSQ total score from all assessments>0	Limited contribution to model
Food security score	Mean score			Mean FSQ total score from all assessments	Limited contribution to model
Sex	Boys 0, girls 1	YES	3		Boys and girls grow differently; Interaction was necessary only for first 3 periods.
LAZ at enrolment	LAZ	YES	3	If LAZe was missing, replaced with LAZ1. (2 children in Brazil)	How children grow is related to how they started; Interaction was necessary only for first 3 periods.
WAZ at enrolment	WAZ	YES	intercept only	If WAZe was missing, replaced with WAZ1. (2 children in Brazil)	How children grow is related to how they started.
First born child	Yes 1, no 0			If DAFlive==1, child is first born.	Relatively lower relationship with growth and limited number of variables that can be included in the model
Dirt floor	Yes 1, no 0			If FSEfloor==1 (dirt) at any time during follow up	Relatively lower relationship with growth and limited number of variables that can be included in the model
Maternal age category	<21, 21-30, 31+			Categorized maternal age	Relatively lower relationship with growth and limited number of variables that can be included in the model

Site		YES	6		Children grow differently at different sites. Site was included as a fixed effect, as well as interacting with the age splines.
Age	Months	YES			Growth rates change by age.
**ALRI**					
Prevalence	Days			1 if ALRI, 0 if no ALRI. ALRI definition met when 1) Child had cough or shortness of breath (today or yesterday) & 2) Rapid respiration rate today (average of two measurements) as defined by a) >60 when child is <60 days old, b) >50 when child is >60-<365 days of age, and c) >40 when child is >365 days of age. ALRI episodes are separated by 14 ALRI-'free' days. 1 for all days in episode (all days with "ALRI")	Relatively lower relationship with growth and limited number of variables that can be included in the model
Prevalence with antibiotics	Days			Only days in episodes coincident with antibiotic treatment at any time during episode	
Prevalence without antibiotics	Days			Only days in episodes NOT coincident with antibiotic treatment at any time during episode	
Incidence	Episodes			1 if first day of episode, 0 if not (each episode counted once at beginning of episode)	
Incidence with antibiotics	Episodes			Only episodes coincident with antibiotic treatment at any time during episode	
Incidence without antibiotics	Episodes			Only episodes NOT coincident with antibiotic treatment at any time during episode	
**Diarrhea**					

Prevalence	Days			Diar=1 if numls>2 or if [safblood==1 and the child had 1 to 2 loose stools]. Days when the study definition is met, but there was an LM test that day or the previous day, were set to 0 (not 'diarrhea' since LM has been found to cause loose stools). 1 for all days in episode (all days with "diarrhea").	Relatively lower relationship with growth and limited number of variables that can be included in the model
Prevalence with antibiotics	Days			Only days in episodes coincident with antibiotic treatment at any time during episode	
Prevalence without antibiotics	Days			Only days in episodes NOT coincident with antibiotic treatment at any time during episode	
Incidence	Episodes			1 if first day of episode, 0 if not (each episode counted once at beginning of episode)	
Incidence with antibiotics	Episodes			Only episodes coincident with antibiotic treatment at any time during episode	
Incidence without antibiotics	Episodes	YES	6	Only episodes NOT coincident with antibiotic treatment at any time during episode	Diarrhea was a key risk factor in the design of this study. Lowest AIC was found when diarrhea without antibiotic treatment was included.
Prevalence of persistent diarrhea	Days			Duration >14 days	Relatively lower relationship with growth and limited number of variables that can be included in the model
Prevalence of severe diarrhea	Days			Severity score >3	
Incidence of persistent diarrhea	Episodes			Duration >14 days	
Incidence of severe diarrhea	Episodes			Severity score >3	

Other illness

Fever	Days			Maternal report of fever, 1=yes, 0=no	Relatively lower relationship with growth and limited number of variables that can be included in the model
Cough	Days			Maternal report of cough, 1=yes, 0=no	
Vomiting	Days			Maternal report of vomiting, 1=yes, 0=no	
Ill (cough, fever, diarrhea or ALRI)	Days			Illness variable indicates whether diarrhea episode, cough, fever, or vomiting was present on that day. (1 if dprev3==1, safcough==1, saffev==1, safvom==1)	

Pathogens

Density	Pathogens/ stool	YES	6	Average number of pathogens in monthly samples without nrv	In order to include diarrhea in the model, pathogen density from non-diarrheal (surveillance) stools were included.
Viruses	Any			Any viruses detected in surveillance stool samples	Relatively lower relationship with growth and limited number of variables that can be included in the model
Bacteria	Any			Any bacteria detected in surveillance stool samples	
Campylobacter	Any	YES		Any campylobacter detected in surveillance stool samples	Second model was run with top 6 individual pathogens.
lt_ETEC	Any	YES	6	Any lt_ETEC detected in surveillance stool samples	Second model was run with top 6 individual pathogens.

Crypto	Any	YES	6	Any Crypto detected in surveillance stool samples	Second model was run with top 6 individual pathogens.
Giardia	Any	YES	6	Any Giardia detected in surveillance stool samples	Second model was run with top 6 individual pathogens.
EAEC	Any	YES	6	Any EAEC detected in surveillance stool samples	Second model was run with top 6 individual pathogens.
aEPEC	Any	YES	6	Any aEPEC detected in surveillance stool samples	Second model was run with top 6 individual pathogens.
**Breastfeeding**					
Full breastfeeding >95% days	Yes 1, no 0	YES	1	Binary, 1 if full breastfeeding % >95%	Relatively lower relationship with growth and limited number of variables that can be included in the model
Full breastfeeding or partial breastfeeding plus dairy >95% days	Yes 1, no 0	YES	1	Binary, 1 if full breastfeeding or partial breastfeeding plus dairy % >95%	
Intake of any dairy products >95% days	Yes 1, no 0	YES	1	Binary, 1 if dairy in diet >95% of days	
**Complementary feeding**					
Energy	Mean kilocalories	YES	3	Average intake of kcal reported in diet during period	Calories in 9-11, 12-17, and 18-24m included, representing caloric content of complementary food.

Protein residuals	Mean residuals	YES	3	Values were transformed (square root) and adjusted for energy using the residual method	Protein residuals represent the energy adjusted protein intake. Protein residuals were highly correlated with micronutrient residuals, which also appeared to be important in the model.
Iron residuals	Mean residuals			Values were transformed (square root) and adjusted for energy using the residual method	Correlation with protein residuals
Vitamin B12 residuals	Mean residuals			Values were transformed (square root) and adjusted for energy using the residual method	Correlation with protein residuals
Vitamin B6 residuals	Mean residuals			Values were transformed (square root) and adjusted for energy using the residual method	Correlation with protein residuals
Zinc residuals	Mean residuals			Values were transformed (square root) and adjusted for energy using the residual method	Correlation with protein residuals
Vitamin C residuals	Mean residuals			Values were transformed (square root) and adjusted for energy using the residual method	Correlation with protein residuals
Vitamin A residuals	Mean residuals			Values were transformed (square root) and adjusted for energy using the residual method	Correlation with protein residuals
Folate residuals	Mean residuals			Values were transformed (square root) and adjusted for energy using the residual method	Correlation with protein residuals
Calcium residuals	Mean residuals			Values were transformed (square root) and adjusted for energy using the residual method	Correlation with protein residuals

Diversity			
Minimum meal frequency	% of months	0/1; Consumption of minimum meal frequency based on WHO core indicator definition. "1" if breastfed child recieved 2 meals and non-breastfed child recieves 4 meals.	Relatively lower relationship with growth and limited number of variables that can be included in the model
Minimum diet diversity	% of months	0/1; Consumption of minimum dietary diversity, which is defined as child consuming 4 or more food groups in the previous day.	
Diversity score	Mean, 0-6	0-6; 6 is the highest diversity score a child could receive if he/she consumes breastmilk, 4 or more food groups, feeds at least 5 times and does not consume formula in the previous day (for children 6-8 months)	
Sum of food groups	Mean, 0-7	0-7 = Sum of food groups consumed based on WHO diet diversity for all children. The 7 foods groups used for tabulation of this indicator are: grains, roots and tubers, legumes and nuts, dairy products (milk, yogurt, cheese), flesh foods (meat, fish, poultry and liver/organ meats), eggs, vitamin-A rich fruits and vegetables, and other fruits and vegetables	
Minimum meal frequency >50% of time	Yes 1, no 0	Binary, 1 if fed minimum meal frequency >50% of period	
Minimum diet diversity >50% of time	Yes 1, no 0	Binary, 1 if fed minimum diet diversity >50% of period	
Animal source protein >50% of time	Yes 1, no 0	Binary, 1 if fed animal source protein >50% of period	

Supplemental Table 3: Values underlying Figure 1, the predicted difference in length, LAZ, weight, and WAZ at 24 months between scenarios that alter potential risk factors and a scenario based on the average experience of children in the cohort. Absolute length and weight were converted into Z-scores using the WHO Growth Standards. The high and low scenarios are based on changing the named variable(s) to the 90th and 10th percentiles of their observed distribution respectively whilst holding all other variables at their mean level. BF refers to % days of full breastfeeding from 0-5 months, and complementary food refers to the % of days fed animal milks and dairy between 3 and 8 months, and energy and (energy-adjusted) protein intakes from non-breast milk foods from 9-24 months.

**Scenario**	**Boy**				**Girl**			
	**Length (cm)**	**LAZ**	**Weight (kg)**	**WAZ**	**Length (cm)**	**LAZ**	**Weight (kg)**	**WAZ**
Average	82.3 (82.1, 82.5)	-1.8 (-1.9, -1.7)	10.9 (10.8, 11)	-1.0 (-1.0, -0.9)	81.2 (81, 81.5)	-1.6 (-1.7, -1.5)	10.3 (10.2, 10.4)	-0.9 (-1.0, -0.8)
Low Diarrhea	82.4 (82.1, 82.6)	-1.8 (-1.9, -1.7)	10.8 (10.7, 10.9)	-1.0 (-1.1, -1.0)	81.3 (81, 81.6)	-1.6 (-1.7, -1.5)	10.3 (10.1,10.4)	-0.9 (-1.1, -0.8)
High Diarrhea	82.0 (81.7, 82.4)	-1.9 (-2.0, -1.8)	10.9 (10.7, 11)	-1.0 (-1.1, -0.9)	81 (80.6, 81.4)	-1.7 (-1.8, -1.6)	10.3 (10.1, 10.5)	-0.9 (-1.1, -0.7)
Low Pathogens	82.8 (82.5, 83.2)	-1.6 (-1.7, -1.5)	10.9 (10.7, 11.1)	-1.0 (-1.1, -0.8)	81.8 (81.4, 82.2)	-1.4 (-1.6, -1.3)	10.3 (10.2, 10.5)	-0.9 (-1.0, -0.7)
High Pathogens	81.6 (81.2, 82.0)	-2.0 (-2.2, -1.9)	10.8 (10.6, 11)	-1.0 (-1.2, -0.9)	80.6 (80.2, 81)	-1.8 (-1.9, -1.7)	10.3 (10.1, 10.4)	-0.9 (-1.1, -0.8)
Low Diarrhea and Pathogens	82.9 (82.5, 83.3)	-1.6 (-1.7, -1.5)	10.9 (10.7, 11.1)	-0.95 (-1.1, -0.8)	81.9 (81.5, 82.3)	-1.4 (-1.5, -1.3)	10.3 (10.1, 10.5)	-0.9 (-1.1, -0.7)
High Diarrhea and Pathogens	81.4 (80.9, 81.9)	-2.1 (-2.3, -1.9)	10.9 (10.6, 11.1)	-1.0 (-1.2, -0.8)	80.3 (79.8, 80.8)	-1.9 (-2.1, -1.7)	10.3 (10.1, 10.5)	-0.9 (-1.1, -0.7)
Low Complementary food	81.6	-2.0	10.5	-1.3	80.6	-1.8	9.9	-1.2

	(81.2, 82.0)	(-2.2, -1.9)	(10.3, 10.6)	(-1.5, -1.2)	(80.2, 81)	(-1.9, -1.7)	(9.7, 10.1)	(-1.4, -1.1)
	83.0	-1.6	11.3	-0.6	81.9	-1.4	10.7	-0.6
High Complementary food	(82.6, 83.4)	(-1.7, -1.4)	(11.1, 11.4)	(-0.8, -0.6)	(81.6, 82.3)	(-1.5, -1.3)	(10.5, 10.9)	(-0.7, -0.4)
	81.8	-2.0	10.5	-1.3	80.7	-1.8	9.9	-1.2
Low BF + Low Complementary food	(81.3, 82.3)	(-2.1, -1.8)	(10.3, 10.7)	(-1.5, -1.1)	(80.3, 81.2)	(-1.9, -1.6)	(9.7, 10.2)	(-1.4, -1.0)
	83.1	-1.5	11.4	-0.6	82.1	-1.3	10.8	-0.5
High BF + High Complementary food	(82.6, 83.6)	(-1.7, -1.4)	(11.2, 11.6)	(-0.7, -0.4)	(81.6, 82.6)	(-1.5, -1.2)	(10.6, 11)	(-0.7, -0.3)
Low Diarrhea and Pathogens	83.7	-1.3	11.3	-0.6	82.6	-1.2	10.7	-0.6
+ High Complementary food	(83.2, 84.2)	(-1.5, -1.2)	(11.1, 11.5)	(-0.8, -0.5)	(82.1, 83.1)	(-1.3, -1.0)	(10.5, 10.9)	(-0.7, -0.4)
High Diarrhea and Pathogens	80.7	-2.3	10.4	-1.4	79.7	-2.1	9.9	-1.2
+ Low Complementary food	(80.1, 81.3)	(-2.5, -2.1)	(10.2, 10.7)	(-1.5, -1.1)	(79.1, 80.3)	(-2.3, -1.9)	(9.6, 10.1)	(-1.5, -1.1)

Supplemental Table 4: Values underlying Figure 2, the predicted difference in length, LAZ, weight, and WAZ at 24 months between scenarios that alter potential risk factors and a scenario based on the average experience of children in the cohort. Absolute length and weight were converted into Z-scores using the WHO Growth Standards. The high and low scenarios are based on presence or absence of the named pathogen in at least one surveillance stool in each period whilst holding all other variables at their mean level. The pathogens represented here are the top three pathogens by prevalence (*Campylobacter*, EAEC, *Giardia*).

**Scenario**	**Boys**				**Girls**			
	**Length (cm)**	**LAZ**	**Weight (kg)**	**WAZ**	**Length (cm)**	**LAZ**	**Weight (kg)**	**WAZ**
Average	82.3<br>(82.0, 82.5)	-1.8<br>(-1.9, -1.7)	10.9<br>(10.8, 11.0)	-0.9<br>(-1.0, -0.9)	81.2<br>(81.0, 81.5)	-1.6<br>(-1.7, -1.5)	10.3<br>(10.2, 10.4)	-0.9<br>(-0.9, -0.8)
High Campylobacter	81.6<br>(81.1, 82.1)	-2.0<br>(-2.2, -1.9)	10.7<br>(10.5, 10.9)	-1.1<br>(-1.3, -0.9)	80.6<br>(80.0, 81.1)	-1.8<br>(-2, -1.7)	10.1<br>(9.9, 10.4)	-1.0<br>(-1.2, -0.8)
High EAEC	81.8<br>(81.3, 82.2)	-2.0<br>(-2.1, -1.8)	10.8<br>(10.7, 11)	-1.0<br>(-1.1, -0.9)	80.7<br>(80.3, 81.1)	-1.8<br>(-1.9, -1.6)	10.3<br>(10.1, 10.4)	-0.9<br>(-1.1, -0.8)
High Giardia	82.2<br>(81.7, 82.7)	-1.8<br>(-2, -1.7)	10.9<br>(10.7, 11.1)	-0.9<br>(-1.1, -0.8)	81.1<br>(80.6, 81.7)	-1.6<br>(-1.8, -1.5)	10.3<br>(10.1, 10.6)	-0.9<br>(-1.1, -0.7)
High Pathogens	81.0<br>(80.3, 81.8)	-2.2<br>(-2.5, -2.0)	10.6<br>(10.3, 11.0)	-1.2<br>(-1.5, -0.9)	80.0<br>(79.2, 80.7)	-2.0<br>(-2.2, -1.8)	10.0<br>(9.7, 10.4)	-1.1<br>(-1.4, -0.8)
Low Campylobacter	82.4<br>(82.1, 82.8)	-1.8<br>(-1.9, -1.6)	10.9<br>(10.8, 11.1)	-0.9<br>(-1.1, -0.8)	81.4<br>(81.0, 81.7)	-1.6<br>(-1.7, -1.4)	10.3<br>(10.2, 10.5)	-0.9<br>(-1.0, -0.7)
Low EAEC	82.6<br>(82.2, 83.0)	-1.7<br>(-1.8, -1.6)	10.9<br>(10.8, 11.1)	-0.9<br>(-1.1, -0.8)	81.6<br>(81.2, 81.9)	-1.5<br>(-1.6, -1.4)	10.4<br>(10.2, 10.5)	-0.8<br>(-1.0, -0.7)
Low Giardia	82.4<br>(82.1, 82.6)	-1.8<br>(-1.9, -1.7)	10.9<br>(10.8, 11.0)	-1<br>(-1.1, -0.9)	81.3<br>(81.0, 81.6)	-1.6<br>(-1.7, -1.5)	10.3<br>(10.2, 10.4)	-0.9<br>(-1.0, -0.8)
Low Pathogens	82.9<br>(82.4, 83.3)	-1.6<br>(-1.8, -1.5)	10.9<br>(10.7, 11.1)	-0.9<br>(-1.1, -0.8)	81.8<br>(81.4, 82.3)	-1.4<br>(-1.6, -1.3)	10.3<br>(10.1, 10.5)	-0.9<br>(-1.0, -0.7)

Supplemental Table 5. Comparison of children who were excluded and included in the analysis. Comparison of continuous variables was performed using t-tests, and of binary variables was performed using chi-squared tests. Asterisks indicate if groups were statistically significantly different after Bonferroni correction for multiple comparisons.

1. Comparison of Z-scores at enrolment

Site	# Excluded	# Included	LAZe, excluded	LAZe, included	WAZe, excluded	WAZe, included	WLZe, excluded	WLZe, included
BG	59	206	**-1.48	-0.98	-1.46	-1.27	-0.61	-1.01
BR	152	81	-0.82	-0.85	-0.23	-0.14	0.33	0.60
IN	51	200	-1.28	-0.97	-1.43	-1.27	-1.05	-1.17
NP	32	208	-1.00	-0.67	-1.28	-0.87	-1.11	-0.88
PE	105	198	-0.93	-0.94	-0.60	-0.63	-0.03	-0.05
SA	115	199	-0.91	-0.69	-0.58	-0.37	-0.09	-0.01
TZ	63	199	-1.21	-0.99	-0.25	-0.14	0.69	0.72

** P<0.01

2. Comparison of WAMI, maternal age and maternal education

Site	# Excluded	# Included	Mean WAMI, excluded	Mean WAMI, included	Mean maternal age, excluded	Mean maternal age, included	Years maternal education, excluded	Years maternal education, included
BG	59	206	0.53	0.54	17.47	17.47	4.59	4.53
BR	152	81	0.82	0.82	18.05	17.72	9.38	8.68
IN	51	200	0.50	0.47	20.12	19.62	7.16	6.91
NP	32	208	0.72	0.70	21.22	21.39	9.19	8.07
PE	105	198	0.62	0.61	18.49	17.76	8.05	7.64
SA	115	199	0.75	0.78	21.12	20.09	10.58	10.08
TZ	63	199	0.30	0.31	19.95	20.36	5.41	5.02

3. Comparison of % first born, girls, and mothers with at least a primary education

Site	# Excluded	# Included	% First-born, excluded	% First-born, included	% Girls, excluded	% Girls, included	% mothers with primary education, excluded	% mothers with primary education, included
BG	59	206	51	38	59	49	59	53
BR	152	81	34	32	*55	36	*97	88
IN	51	200	*53	30	53	56	73	74
NP	32	208	**75	40	44	46	91	80
PE	105	198	41	37	54	43	87	88
SA	115	199	42	37	53	49	99	99
TZ	63	199	21	10	52	50	73	68

* P<0.05
** P<0.01
